# The Role of Glycans in Bacterial Adhesion to Mucosal Surfaces: How Can Single-Molecule Techniques Advance Our Understanding?

**DOI:** 10.3390/microorganisms6020039

**Published:** 2018-05-04

**Authors:** Cécile Formosa-Dague, Mickaël Castelain, Hélène Martin-Yken, Karen Dunker, Etienne Dague, Marit Sletmoen

**Affiliations:** 1 LISBP, Université de Toulouse, CNRS, INRA, INSA, 31400 Toulouse, France; formosa@insa-toulouse.fr (C.F.-D.); castelai@insa-toulouse.fr (M.C.); helene.martin@insa-toulouse.fr (H.M.-Y.); 2 Department of Biotechnology and Food Science, NTNU the Norwegian University of Science and Technology, NO-7491 Trondheim, Norway; karen.dunker@ntnu.no; 3 LAAS-CNRS, Université de Toulouse, CNRS, 31400 Toulouse, France; edague@laas.fr

**Keywords:** carbohydrate recognition, mucus, adhesins, lectins, mucus adhesion, glycan interactions, AFM, optical tweezers

## Abstract

Bacterial adhesion is currently the subject of increased interest from the research community, leading to fast progress in our understanding of this complex phenomenon. Resent research within this field has documented the important roles played by glycans for bacterial surface adhesion, either through interaction with lectins or with other glycans. In parallel with this increased interest for and understanding of bacterial adhesion, there has been a growth in the sophistication and use of sensitive force probes for single-molecule and single cell studies. In this review, we highlight how the sensitive force probes atomic force microscopy (AFM) and optical tweezers (OT) have contributed to clarifying the mechanisms underlying bacterial adhesion to glycosylated surfaces in general and mucosal surfaces in particular. We also describe research areas where these techniques have not yet been applied, but where their capabilities appear appropriate to advance our understanding.

## 1. Introduction

Recent research papers illustrate that bacterial adhesion is currently the subject of increased interest from the research community, leading to fast progress in our understanding of this complex phenomenon. In many of these papers, glycans are recognized as essential for the adhesion, either through interaction with lectins or with other glycans. The increased awareness of the numerous and important functions of glycans in biological systems, including but not limited to bacterial adhesion, recently motivated researches to conclude that “*glycoconjugates, and glycoproteins in particular, are an underappreciated, potentially crucial, factor in understanding bacteria*–*host interactions*” [1]. In parallel with the increased awareness of the functions of glycans in biological systems, we have witnessed an increased awareness of the importance of mechanical processes occurring in living cells [2]. As pointed out by Carlos Bustamante more than a decade ago, mechanical processes are involved in nearly every facet of the cell cycle, including but not limited to chromosomal segregation, replication, transcription, translation, translocation of proteins across membranes and cell locomotion. In addition to these examples of intracellular processes, mechanical processes are essential for cellular and bacterial adhesion to surfaces. The ability to directly apply external forces to cells, cellular components or single molecules, through the use of force probes, is therefore a powerful approach that can greatly contribute to revealing the underlying molecular mechanisms. 

The increased awareness of the importance of forces for biological systems has been aided by a growth in the sophistication and use of sensitive force probes for single-molecule and single cell studies. This field includes studies in which atomic force microscopy (AFM) is used to study the adhesive properties of microbes, as recently reviewed [3]. In this review, Dufrêne provides an interesting introduction to the forces driving cell adhesion and biofilm formation as revealed through AFM studies, but without directly addressing the role of glycans in this adhesion. However, the sensitive force probes AFM and optical tweezers (OT) are well-suited tools to study glycan-based interactions. This is thus a suitable time to review how these sensitive force probes have contributed to clarifying the mechanisms underlying bacterial adhesion to glycosylated surfaces in general and mucosal surfaces in particular. We also describe research areas where these techniques have not yet been applied, but where their capabilities appear appropriate to advance our understanding. 

## 2. Sensitive Force Probes and Their Use for Single Molecule and Single Cell Interaction Studies 

### 2.1. Optical Tweezers (OT): Working Principle and Applicability for Studies of the Mechanisms Underlying Microbial Adhesion

**Figure 1:**
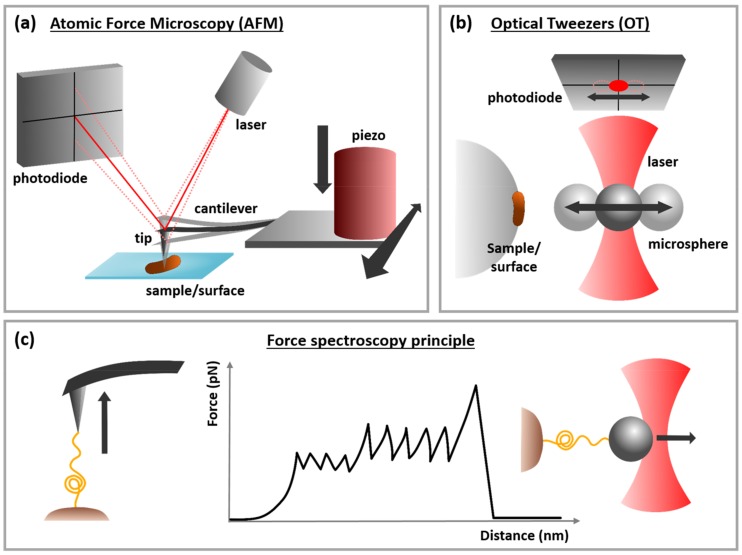
Atomic force microscopy, optical tweezers and force spectroscopy principles. (**a**) AFM principle: a sharp tip is mounted on a cantilever that can be moved in the x, y, and z direction thanks to a piezo-electric ceramic. A laser beam reflected on a photodiode allows recording the deflection of the cantilever. Reproduced with permission from [33]. (**b**) OT principle: A small bead is trapped in the waist of a focused laser beam. The beam transmitted/refracted by the trapped bead is reflected on a photodiode allowing to monitor the position of the small bead from the focus position. A large bead, on which a living cell is grafted, is immobilized on the microscope slide; an x, y, and z piezo stage moves it towards the trapped small bead in order to interact. (**c**) Force spectroscopy principle: both in AFM and OT, the probe interacts with a molecule present at the surface of the sample, and then is retracted from the sample, which leads to the unfolding of this molecule. When the retraction force is high enough that a molecular bond within the molecule is broken, a retract adhesion peak appears on the force curve (middle scheme). When several intramolecular bonds are broken successively, several retract adhesion peaks are visible on the force curve. The force needed to break the bond can be measured directly on the force curve, as well as the length of the unfolded molecule.

Since the invention of the OT technique in 1970 by Arthur Ashkin [4] in the Bell lab, microbial adhesion, mainly driven by molecular interactions that can be probed using OT, gained a large interest from biophysicists. Ashkin and co-workers [5,6] pioneered the use of light to micromanipulate micro-sized particles by exerting pico-Newton (pN) forces in the waist of a highly focused laser beam by a high numerical aperture microscope objective (Figure 1b). This technique opened a large avenue for force spectroscopy as it made it possible to both pull single molecules such as DNA [7], titin [8], or pili [9] with forces in the pN-range and also to determine the molecular interactions driving adhesion processes. Since OT allows reliable quantification of forces at the sub-pN level, many studies on bacterial adhesion have been performed at the single cell level by either manipulating the cell itself [10,11,12,13] or a functionalized trapped bead that can interact with an immobilized cell [9,14,15,16,17,18]. This latter configuration allowed determining important biophysical molecular parameters such as bond lifetime, bond length or thermal off-rate [19]. These parameters were then conceptualized by Bell [20] and then Evans [21], who proposed an experimental approach called dynamic force spectroscopy (DFS), illustrated in Figure 1c, as well as a model to describe the cell adhesion through energy landscapes [21]. In DFS, single-molecule techniques such as AFM [22,23,24,25,26] and OT [27,28,29,30] are used to scrutinize molecular interactions and conformations and determine their energy landscapes. For this approach, DFS analyses the failure of bonds formed between single pairs of molecules when exposed to external force. For instance, Evans et al. in [31] found that the typical rupture force of a single bond is proportional to the logarithm of the loading rate when it is driven out of the equilibrium. Note that this is the case when the applied force increases linearly with time so that the bio-complex is exposed to a constant loading rate. Interestingly, Björnham and Andersson have recently formulated nonlinear DFS for molecules such as tethers or polymers that are able to modulate the applied force in a nonlinear manner and validated their model with both experimental data obtained using force-measuring OT and Monte-Carlo simulations [32].

### 2.2. Atomic Force Microscopy (AFM): Working Principle and Applicability for Studies of the Mechanisms Underlying Microbial Adhesion 

Atomic force microscopy (AFM) was first developed in 1986 [34] and relies on the control of the force acting between a sharp tip, usually made of silicon nitride, and the surface while scanning a sample (Figure 1a). Especially in microbiology, its high-resolution imaging capacities have attracted many scientists who have used this technology to image the structure and ultrastructure of various types of bacterial cells [33,35,36,37]. However, AFM is not only an imaging technique, it is also a sensitive force probe AFM uses the same theoretical framework as used in OT studies (Figure 1c). In thee force curves, the force experienced by the probe is presented as a function of the tip-sample separation distance. Whereas the approach curve gives quantitative information on the sample height or its mechanical properties, the retract curve contain information related to the adhesive forces acting between the AFM tip and the surface of the sample [38]. By collecting a matrix of force distance (FD) curves across a sample surface, the information can be spatially resolved. This is referred to as force volume imaging, spatially resolved force spectroscopy, affinity mapping or also molecular recognition imaging [39,40]. Recently, technological advances have made it possible to record FD curves at a high frequency so that adhesion, height and mechanical maps of samples can be acquired with high spatiotemporal resolution. Force-volume imaging thus allows to simultaneously image biological samples and quantify their intrinsic biophysical properties [41,42,43]. In a recent study, such multiparametric measurements revealed the unexpected influence of zinc ions on the activation of a cell-adhesion protein of *Staphylococcus aureus*. The presence of zinc ions was found to induce modifications of the surface structure of the cells, but also of the nanomechanical and adhesive properties of their cell wall [44]. Another possibility that underlies much of the interest for this technique is the possibility to work with functionalized AFM tips. As further explained in the next section, AFM tips, as well as surfaces used in OT experiments, can be functionalized in many different ways. As a result of these surface modifications the surface of the tip carries well-defined chemical groups [45], nanoparticles [46,47], biological molecules such as proteins or antibodies [48], or living cells [49]. The strength of adhesive interactions formed between such functionalized tipa and the sample can be quantified using FD curves and force-volume AFM enables mapping of these interactions across the sample surface. We will in the next paragraph focus on tip functionalization strategies involving biomolecules and living cells, given their relevance to understand the role of glycans in bacterial adhesion to mucosal surfaces. 

### 2.3. Strategies Used to Functionalize Surfaces Used in OT or AFM Studies

When using OT or AFM to study specific or non-specific adhesive interactions formed between two interacting surfaces, it is often necessary to functionalize the surfaces with molecules of interest, cells or biological structures, for example pili. For AFM studies the molecules or cells are immobilized either on the AFM tip, or on a flat surface that the tip is allowed to approach and retract from. For OT studies, the molecules or biological structures of interest are usually immobilized onto silica or polystyrene microspheres ranging in size from 1 to a few micrometers in diameter. Bacteria can also be trapped directly by the laser trap. 

The easiest way to immobilize biomolecules onto a surface is by physical adsorption. However, this technique is non-specific, which might influence the significance of the results obtained. In the case of AFM, an alternative strategy involves chemical fixation of molecules via sulfur-gold bonds to gold-coated AFM tips, using self-assembled alkanethiol monolayers. For example, for protein immobilization, the gold tip can be functionalized with a carboxyl- or amino-terminated thiol, that can be activated by 1-ethyl-3-(dimethylaminopropyl) carbodiimide hydrochloride (EDC), and *N*-hydroxysulfosuccinimide (NHS). Proteins or other molecules of interest can be linked to this carboxyl-terminated thiol by the formation of amide bonds between the activated carboxyl groups and the amine groups present on the proteins (or vice versa) [50]. This type of strategy has been used in many studies involving bacterial attachment; for example, Valotteau and co-workers used it to determine the cell surface ligands of the collagen-binding protein (Cna) of the bacterial pathogen *S. aureus* [51]. Alternative immobilization strategies, that give rise to covalent attachment of molecules onto silicon nitride AFM tips or commercially available silica or polystyrene beads that carry reactive surface groups, are also available. These approaches require that reactive groups are first introduced on the surface of the AFM tip or beads used in OT experiments. Amino-groups can be introduced for example through esterification with ethanolamine or silanization with aminopropyl-triethoxysilane (APTES). A range of different chemical groups can be introduced by the use of commercially available silanes with the suitable structure. Proteins or other biological structures can then be coupled covalently to these surfaces through chemical reactions between the reactive chemical groups on the functionalized AFM tip or bead, and reactive groups on the molecule or sample to be immobilized. An often used reaction is the Schiff base formation between an amine and a carbonyl group [50]. Biomacromolecules can also be immobilized onto amino-terminated surfaces through heterobifunctionalized polyethylene glycols (PEG) [52,53], or aldehyde-phosphorus dendrimers [54]. The use of these methods allows recording specific interactions between proteins or other molecules of interest grafted onto AFM tips or microbeads. Studies in which the interactions between two immobilized molecules are quantified as a function of the force loading rate are named dynamic force spectroscopy (DFS) or single-molecule force spectroscopy (SMFS) studies. 

If a living cell is immobilized onto one or both of the interacting surfaces, these sensitive force probes enable single-cell force spectroscopy (SCFS) experiments, and thus allow the direct monitoring of interactions between two cells or between a cell and a surface [55]. Pioneer work on immobilization of bacteria on AFM tips was performed by Razatos and co-workers in 1998, who immobilized *Escherichia coli* cells on polyethylenimine (PEI) coated AFM tips [56]. Following this work, many strategies were developed to immobilize single or multiple bacterial cells on AFM cantilevers, using for example cantilevers coated with charged polymers [57] or silanes [58], hydrophobic alkanelthiols [59], or bioinspired wet adhesives [60]. For such work, tip-less cantilevers are often used. To generate probes functionalized with only one bacterial cell, given the small size of bacterium compared to the cantilever, it is for some studies preferable to first attach a micrometer-sized colloidal particle to the cantilever. This colloidal probe is then coated with a wet adhesive, such as polydopamine, which allows adhesion of single live cells [49]. More recently, with the emergence of fluidic force microscopy (FluidFM) [61], a new method to immobilize single bacterial cells without using chemicals was made available. This AFM based approach aims at aspirating a single cell at the end of a pyramidal microchanneled AFM cantilever, driven by the underpressure generated by a microfluidic system [62]. One advantage of this method is that no molecules are used to attach the bacteria, so the bacterial interface remains unchanged. Another advantage is that the cell immobilization is reversible, thus allowing testing many cells in a single experiment, an approach that greatly facilitates data collection as needed for statistical analysis. 

These recent years, both SMFS and SCFS have been used to understand the complex molecular dynamics involved in bacterial attachment, either to surfaces, or to other cells. We will later in this review present examples of studies where such techniques are used to characterize and quantify the forces driving adhesion of bacteria to mucosal surfaces, and more specifically we will see how such techniques have contributed to understand the role of glycans in theses interactions. 

## 3. Mechanisms of Glycan-Based Adhesion of Bacteria

The molecular mechanisms of adhesion are for many bacteria and surfaces not well known. In the following, we will present systems where evidence that allow identifying the main molecular pair interactions that contribute to the adhesion is available, and where glycans are essential for the adhesion. The identification of the molecules that drive the adhesion is for many systems challenging, since more than one mechanism of interaction may play a part. Furthermore, the relative importance of each of the mechanisms may change over time and between different adherent surfaces, as documented in the literature [57]. Nevertheless, in order to understand and control bacterial adhesion it is essential to identify the main contributions to the adhesion for each bacterium-surface system as well as its properties (strength, responsiveness to changes in environmental parameters, etc.).

### 3.1. General Considerations about the Mechanisms Underlying Microbial Adhesion to Mucosal Surfaces 

Many internal surfaces in both humans and animals are covered by mucus, which lines the cells to protect the host from external threat [63,64]. That is why mucus is found in the respiratory tract [65], vaginal cavity [66] and urinary tract [67]. The gut is also covered by a mucus layer that plays an important role in immune regulation and hosts the gut microbiota [68]. Mucins are highly glycosylated glycoproteins. They have the ability to form fibers that can be organized in a network (see for example the extensive review of Bansil and Turner [69]), which confers to the mucus non-Newtonian viscoelastic shear-thinning properties [70,71,72], meaning that its viscosity decreases with increasing shear rate. The primary function of mucus is to act as a filter, permitting the selective passage of components that are beneficial to the body while serving as a barrier against potentially harmful ones, such as viruses and pathogens. Understanding the filter properties of the mucosal barrier has gained significant interest due to its relevance for the development of novel drug delivery strategies, such as nanoparticle application systems [73]. The diffusion through this hydrogel is hindered for given particles and allowed for others [74], working as an interaction selective filter [75]. Mucus is mainly composed of MUC proteins. A number of mucin genes have been described, each giving rise to a specific sub-family of mucins. Different mucins predominate in different tissue, e.g., MUC2 for small intestine [76], and MUC5AC mainly in the gastric region [77] (see an exhaustive reference list in the review of Lindén et al. [78]). It has been argued that, in addition to the protective role of the mucosa, mucins harbor glycan-rich domains that provide preferential binding sites for pathogens and commensal bacteria [79]. Due to different adhesion abilities of bacteria to the mucosa, they are either trapped in the mucosal net or diffuse more or less freely to reach the host cells. This has been observed with *H. pylori* that is able to swim through the gastric mucosa by decreasing its viscosity with urease activity and pH increase [80,81]. Once they reach the host gastric cells, the *H. pylori* bacteria adhere to fucosylated ABO/Lewis b blood group antigen receptors using BabA adhesins fucosylated ABO/Lewis b blood group antigen [82], as further discussed below.

However, microbial adhesion has been dissected into several mechanisms that act either in parallel or in a consecutive manner. These include non-specific interactions, for which the description is strongly inspired by colloidal disciplines such as physicochemical approaches with DLVO theory [83,84,85,86] to the specific side, driven by the ligand-receptor mechanisms [14,87,88] including glycan-lectin interactions. In most cases, bacteria initially adhere to host cells through the action of adhesins that bind specifically to receptors at the surface of host cells. These ligand-receptor interactions have a given strength, presumably optimized for their environment and weak enough to allow a bacterium to detach regularly and migrate to other locations. Interactions can be described by a bond and an energy landscape where the transition between the different states of the bond (open or closed) can be governed by activation energy and bond length [20]. Application of a force on the bond, for example through the application of the sensitive force probes described in Section 2, affects the activation energy and the bond length and gives the opportunity to map out its energy landscape. 

Carbohydrate-binding proteins, named lectins, are widespread in nature and known to be important for microbial adhesion. Well-known in plants since 1888 [89], they are in fact present in almost all cell types which use their exquisite ability to bind specific oligosaccharides motifs, either in solution or on the surface of other cells. Hence, they play key roles in extremely diverse biological processes, from seed germination to flocculation, immune system and cancer development. Their involvement in the adhesion process of microbial cells such as bacteria and fungi to either other microbial cells or to eukaryotic mucosal surfaces has been established since the late seventies [90]. The bacterial lectins are typically in the form of elongated submicroscopic multi-subunit protein appendages, known as fimbriae or pili [91]. Over the last couple of decades, single molecule techniques have been applied to the investigation of these lectins-mucins interactions, and these studies have provided new insight into the molecular mechanisms involved. Additionally, new studies published over the last decade have documented the importance also of glycan-glycan interactions for bacterial adhesion, as well as the importance of the surface organization of the adhesive molecules into islands or patches of increased surface density compared to the surrounding surface. In the following, we review a range of central studies within this area. 

### 3.2. Pili-Based Attachment to Mucosal Surfaces

Pili and fimbriae are hair-like appendages found on the surface of many bacteria. Numerous studies have provided evidence that host glycoconjugates are a common target for several bacterial adhesins that are present on pili and fimbriae [92]. Indeed, recent studies have provided evidence for the lectin activity of pneumococcal pilin proteins [93] as well as sialylation of the tip PilA, a pilus-associated adhesin of *Streptococcus agalactiae* adhesin, and its possible influence on the interactions with host cells [94]. Moreover, in *Lactococcus lactis*, pili and surface proteins have been shown to be involved in interactions with mucus [95]. Bacterial cells can also carry at their surface mucus-binding proteins (MUB) that specifically recognize mucins [96]. The properties of these proteins have been investigated experimentally using different methods such as shear-stress flow chamber [97], AFM [57,98], or QCM-D [99] as further described in Section 3.3. Compared to MUB, pili are more extended structures and thus are better suited to overcome the energy barrier between two surfaces. Le et al. [97] measured, using AFM, rupture distances around 600 nm with pili and 200 nm with MUB. Pili-based attachment is thereby optimized by the length of the structure and by specific ligand recognizing glycans, as reported previously with *Lactobacillus rhamnosus* GG [100]. In this study, the authors obtained AFM retract force curves showing a step-like shape, leading to the conclusion that the detachment of piliated *Lactobacillus rhamnosus* GG from mucin-coated surfaces was involved in a zipper-like process. Accordingly, they showed that adhesins are distributed along the pilus backbone. This pili structure was also proposed for pili from *Streptococcus pneumoniae* TIGR4 [101,102] and further evidenced by force-measuring optical tweezers experiments [103]. Gram-positive pili, and particularly streptococcal pili (including lactococci) seem to share a common quaternary structure with a linear assembly of subunit that are not fully optimized to dissipate energy from environmental forces [18,34,103] as it is the case for *E. coli* pili [104,105]. Interestingly, pathogenic piliated *E. coli* strains express helix-like pili that dampen the external forces and minimize the load at the tip in interaction with a specific host receptor [105,106,107,108]. More information on this specific topic can be found in a recent review [109].

**Figure 2:**
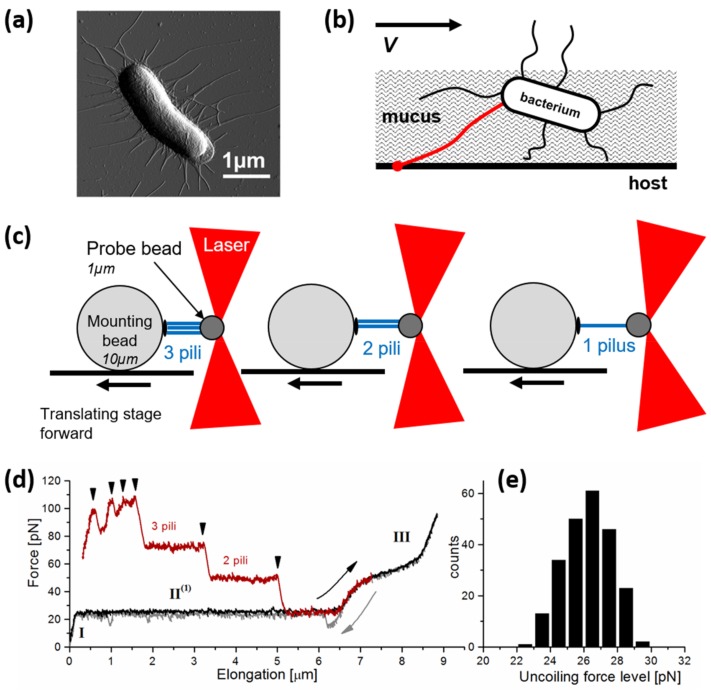
Pili-based adhesions. (**a**) AFM vertical deflection image of an *Escherichia coli* cell expressing F1C pili. Reproduced with permission from [124]. (**b**) Illustration of a bacterial cell embedded in a mucus layer and attached to a host cell, able to withstand a physiological shear flow thanks to its pili. (**c**) A piliated bacterial cell immobilized on a large «mounting bead» interacts through its three pili to a small probe bead trapped by a laser beam. When retracting the stage, the pili are extended and unfolded until one breaks, then another one until the end of the retraction where a single pilus remains, probably the longer one. Adapted with permissions from [34]. (**d**) Force diagram corresponding to the experiment represented in panel (**c**), showing the force-extension curve of F1C pili during their unfolding (force plateaus) in a step-like manner until one single pilus is reached. (**e**) Distribution of the individual unfolding force values reported from the force plateau mean values. Reproduced with permission from [104].

The pili found on *Escherichia coli*, i.e., Type 1 [110,111] and P pili [112,113], have been widely investigated in order to unravel their role in bacterial adhesion [17,18]. Several studies have proposed a relationship between the biomechanics of the surface proteins, when exposed to environmental forces, and adhesive properties. Optical tweezers have been extensively used to quantify the force-dependence of such proteins. For example, Björnham et al. [106] described the energy landscapes of PapG/galabiose complex, obtained from the unwinding of P pili expressed at the surface of uropathogenic *E. coli* (UPEC). PapG is a protein described to be located at the distal end of the P pili and showing an affinity for galabiose. In these studies, an intimate relationship was revealed between the PapG/galabiose complex and the intrinsic biomechanical properties of the helix-like pilus backbone during its extension using OT. Another study focused on FimH, a protein homologous to PapG, present on Type 1 pili expressed on UPEC [114] and known to have specific affinity to mannose. In this study, the interactions between Type 1 UPEC and mannose-presenting surfaces were investigated using OT, and the results obtained provided new insight into the FimH-mediated adhesion of UPEC to mannose-coated surfaces. The force of detachment for a single, monovalent, a-mannoside–pilus interaction was determined to be 1.7 pN. The bacteria were observed to detach in a “Velcro like” process from the mannose-coated surfaces, indicating polyvalent adhesion. The forces to detach bacteria in a side-on (maximal number of adhesive contacts) vs. an end-on orientation (one or two adhesive contacts) were compared and demonstrated the potency of polyvalency in attaining an overall strong attachment to the surface. Later, Andersson et al. [115] addressed the differences between P and type 1 pili biomechanical properties reflected by their environment, a topic that was further investigated by Castelain et al. with other types of pili from UPEC, whose results are presented in Figure 2 [104,116].

FimH at the fimbrial tip is known to bind in a catch-bond mode [117] to terminal a-d-linked mannoses of N-linked glycans urinary epithelial cells [118]. Owing to its important role in establishing infection, FimH is an attractive target for the development of anti-adhesive drugs for treatment of infections including urinary tract infections [91,119]. The properties of this interesting bacterial adhesin have therefore also simulated using both Monte Carlo [120] as well as MD simulations [121]. The catch-bond mechanism of FimH was also recently investigated, and a three-state mechanism of FimH catch-bond formation was suggested based on crystal structure studies, kinetic analysis of ligand interactions and molecular dynamics simulations [122]. In the absence of tensile force, the FimH pilin domain was found to allosterically accelerate spontaneous ligand dissociation from the FimH lectin domain by 100,000-fold, resulting in weak affinity. Stress induced separation of FimH protein domains lead to increased affinity of the lectin domain. The strength of the FimH-mannose interaction was later investigated using AFM in a study where the authors concluded that the interaction strength was in the interval 50 to 75 pN and quasi-independent of the applied pulling force [123]. However, in this study, the loading rate was not determined and this lack of information hinders a proper analysis of the potential catch bond effect. We thus conclude that despite the suitability of sensitive force probes like AFM to study catch-bonds, the properties of the FimH-mannose interaction has to our knowledge so far not been carefully addressed using these techniques.

In addition to all the examples mentioned above, the type IV secretion pathway also plays a major role for bacterial attachment to host cells and for twitching motility [125,126]. The process of twitching motility is governed by the dynamical polymerization/depolymerization of subunit proteins forming bundle-like pili to tract the bacterium forward. Several studies have described this mechanism [125] and quantified the retraction forces using OT [126,127]. A combination of optical and magnetic tweezers as well as AFM were used by Biais et al. to apply forces on purified Type IV pili (Tfp). The study demonstrated that Tfp subjected to approximately 100 pN of force passes through a reversible conformational transition and forms a structure that is roughly 3 times longer and 40% narrower than the original structure. This force-induced conformation exposes hidden epitopes previously buried in the Tfp fiber [128]. Further insight into the force generation, and the atomic-level characteristics of the force-induced conformation have been sought using steered molecular dynamics (SMD) simulation [129]. The buried pilin a1 domains were found to maintain hydrophobic contacts with one another within the core of the filament, thus contributing to structural stability. For more information on this point, the connections between current knowledge in molecular biology and biophysics of Tfp has been reviewed [130]. This review focuses on Tfp behavior towards different surfaces, monitored using biophysical measurement techniques and strategies, including both OT and AFM. 

### 3.3. Non Pili-Based Interactions 

**Figure 3:**
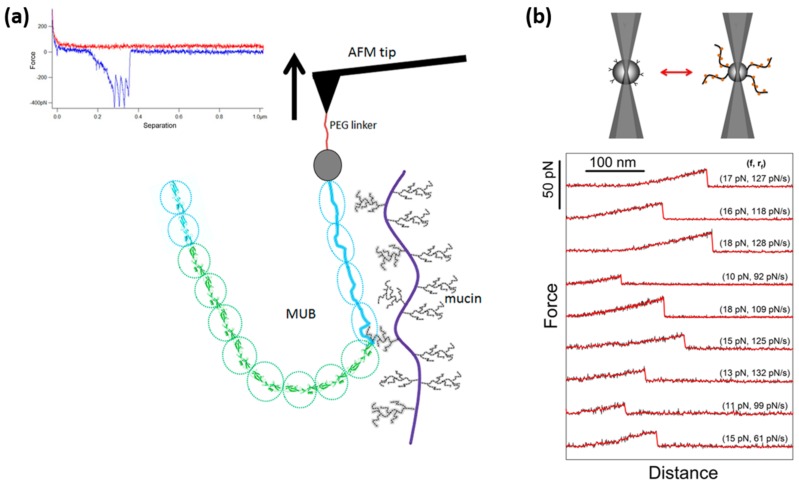
Non-pili based adhesions probed by AFM and OT. (**a**) AFM force curve and schematic representation of the interaction between a mucin-binding protein (MUB) from *L. reuteri* ATCC 53608 and mucins. The force curve shows four retract adhesion peaks corresponding to the unfolding of four type 1 MUB repeat domains (blue domains on the figure). Reprinted with permissions from [131]. (**b**) Schematic illustration of an OT system composed of two optically trapped beads functionalized with MUC1(ST), a mucin harboring α-NeuNAc(2-3)β-Gal(1-3)α-GalNAc-Ser/Thr glycans, and Macrophage galactose/N-acetylgalactosamine (GalNAc) specific lectin (MGL). The force diagram show typical force curves obtained when repeating approach–retract cycles for experimental series of MUC1(ST)–MGL. Reprinted with permissions from [135].

Despite the importance of pili, bacteria also use other mechanisms for adhesion. A recent study by Gunning and coworkers illustrates the strength of sensitive force probes for studying another type of molecular interactions underlying bacterial adhesion. In this study, the nature of the interaction between a purified mucus-binding (MUB) protein and mucins was characterized by FD curve based AFM, as shown in Figure 3a [131]. The canonical MUB protein from *Lactobacillus reuteri* ATCC53608 used in this study displays a long and linear multi-repeat structure. Single-molecule force spectroscopy by AFM allowed these authors to unfold the multiple repeats constituting the adhesin and to show that the interaction between this MUB and mucin followed a nanospring-like adhesion model. In addition, MUB self-interactions also revealed a similar binding pattern. This behavior observed for MUB–mucin interactions was in contrast with their observations for a different family of mucin binding proteins, the mammalian Galectin-3 (Gal-3) The Gal-3-mucin FD curves showed a few occasional multiple adhesive interactions but these were minor in terms of numbers compared to MUB. These observations are in line with the knowledge that Gal-3 is a chimeric lectin containing a single carbohydrate recognition domain (CRD). In addition, the peaks in the Gal-3-mucin retraction curves had relatively large separation distances (sometimes > 100 nm) and lower adhesive magnitude as compared to the MUB–mucin data set. Together, these observations allowed the authors to conclude that the structural organization of MUB maximizes interactions with the mucin glycan receptors through its long and linear multi-repeat structure, potentiating the retention of bacteria within the outer mucus layer. The rare multiple interaction events observed for Gal3-mucin interaction was proposed to be due to the multiplicity of Gal3 binding sites along the mucin molecules [131]. However, as Gal3 is known to organize into pentamer structures, with one binding site on each subunit of the pentamer [132], the rupture strength observed for the Gal3 when interacting with mucin might be due to multiple interactions rupturing within a short time and distance interval, and thus not easily detectable in the FD curve. The degree of multiplicity is, based on the data provided by Gunning and co-workers, lower than what is observed for the MUB–mucin interaction. However, bond multiplicity of the type made possible in interactions involving lectins covalently or non-covalently organized into structures displaying more than one carbohydrate binding site can potentially be of biological importance, and this topic should be further investigated in future studies.

AFM has also been used to investigate the interaction between soybean agglutinin (SBA) and a modified porcine submaxillary mucin (Tn-PSM) decorated with GalNAc residues O-linked to serine or threonine [133]. Unbinding and rebinding events were observed, with rupture distances consistent with the length of the mucin chain. The lifetime of the SBA-TnPSM complex determined in this study was compatible with a binding model in which lectin molecules “bind and jump” from α-GalNAc residue to α-GalNAc residue along the polypeptide chain of Tn-PSM before dissociating. Such a mechanism was first proposed based on data obtained by isothermal calorimetry (ITC) [134]. Remarkably, this description of the mechanism of lectin-mucin binding is reminiscent of protein ligands binding to DNA, which suggests the existence of a common mechanism of ligands binding to biopolymers involving enhanced entropic effects facilitating binding interactions.

Interestingly, AFM has also been used to asses and compare the respective roles of MUB and bacterial pili in pig gastric mucin (PGM) adhesion [97]. In this study, the adhesion of *L. lactis* (TIL448) was investigated both at the molecular level by AFM using cantilevers functionalized with immobilized bacteria as well as at the population scale using a shear stress flow chamber. The use of mutants defective in either MUB or pili allowed the authors to demonstrate that MUB and pili was of comparable importance for the adhesion to PGM under static condition, while MUB contribution was greater than the one of pili under shear flow. Moreover, both short (100–200 nm) and long (600–800 nm) rupture distances were measured by AFM for the *L. Lactis*-PGM interaction, and blocking assays established the key role of neutral oligosaccharides in this binding. 

A similar implication of multiple adhesion mechanisms underlying the observed adhesion of a particular bacterium to a particular surface is also documented in studies focused on other bacteria, using other experimental techniques. For instance, Wang and coworkers used surface plasmon resonance (SPR) to study bacterial adhesion on biomimetic temperature responsive glycopolymer surfaces [136]. These investigations revealed that both hydrophobic and lectin-carbohydrate interactions contribute to the bacterial adhesion to cell surfaces. In another study, the same authors used Quartz Crystal Microbalance with Dissipation (QCM-D) to study lectin-polymer and bacteria-polymer interactions onto glycopolymer-functionalized surfaces [50]. Using this approach, they were able to determine that the lectin *Ricinus communis* agglutinin (RCA120) had a specificity for galactose and to characterize the binding of two bacterial models, *Pseudomonas aeruginosa* PAO1 and *Escherichia coli* K-12 through fimbriae onto different functionalized surfaces. The results obtained revealed a prevalence of lectin–carbohydrate interactions in comparison to nonspecific interactions. 

The high sensitivity of the OT compared to AFM makes this force probe an ideal tool for the quantification of the strength of interactions formed between single molecular pairs of lectins and their carbohydrate ligands. The applicability of OT to quantify the rupture force and properties of single molecular pair interactions formed between lectins and glycans was documented in a study focusing on the binding of C-type lectins to MUC1 mucins displayed at the surface of normal versus cancerous cells, as presented in Figure 3b [135]. Despite this documented applicability of OT in studies aiming at addressing glycan-lectin interactions relevant for bacterial adhesion, examples of such use of OT are to our knowledge lacking. However, studies exist where the interaction between bacteria and functionalized surfaces have been studied, in order to shed light on the properties of surface adhesins. A central study within this field focused on the adhesion of *Helicobacter pylori* to mucosal surfaces [14,19]. More precisely, the authors studied the complex interaction formed between the fucosylated ABO/Lewis b blood group antigen, found in the gastric mucosa and its specific receptor, the BabA adhesin found at the surface of *H. pylori*, well-known to cause gastritis and peptic ulceration. Ruptures forces were determined on single living cells and the data obtained revealed the mechanical properties of the complex as well as the energy landscape of the BabA-ABO/Lewis interactions. The study concluded that the de-adhesion, when measured with a force loading rate of 100 pN/s, ranged from 20 to 200 pN. The de-adhesion force appeared predominantly as multiples of an elementary force, which was determined to 25 ± 1.5 pN and identified as the unbinding force of an individual BabA-Le^b^ binding. The authors concluded that adhesion in general was mediated by a small number of molecular pair interactions, most often 1 to 4, despite the contact surface between the bacterium and the bead encompassing significantly more binding sites. The tendency of the rupture forces to appear as multiples of an elementary force has also been observed in other OT studies of bacterial adhesion. Simpson et al. [11,12,13] used OT to measure the interaction forces of *Staphylococcus epidermis* and *Staphylococcus aureus* when interacting with fibronectin-coated surfaces. The observed binding forces between fibrinogen or fibronectin and *S. aureus* MSCRAMMs occurred as an approximate integer multiple of 20 or 25 pN, respectively, and this was suggested to be related to the formation of multiple bonds [11].

### 3.4. Glycan-Glycan Interactions

All bacterial cells are covered by glycans that play key roles in adhesion to surfaces but also to host mammalian cells, and thus in pathogenesis. The binding partners of bacterial glycans on host cell surfaces have for a long time been considered to be mostly proteins, in the form of adhesins or lectins, as described in Section 3.2 and Section 3.3. However, the body of evidence documenting that glycans engage in direct interactions with other glycans is increasing. Since glycans are known to form a dense coat at the surface of mammalian cells, termed the glycocalyx [137], and also are present in high amounts on the surface of most bacteria, the existence of such interactions open for numerous new mechanisms of bacterial adhesion. Glycan-glycan interactions in these bacterial-mammalian cells systems have been characterized as low-affinity weak interactions that precede high-affinity protein-glycan or protein-protein interactions [138]. However, several recent studies have documented the importance of such interactions in bacterial adhesion. The first report that provides convincing evidence that glycan-glycan interactions can be key interactions for the attachment of bacterial cells to host mammalian cells was published in 2014 by Day and co-workers [139]. In this study, the interactions between lipopolysaccharides (LPS) and lipo-oligosaccharides (LOS) from different human pathogenic bacterial species, and more than 60 host cells glycan structures were measured using glycan microarrays and surface plasmon resonance (SPR) experiments. Their results showed that not only are glycan-glycan interactions in these types of systems widespread phenomena, but they are also high-affinity interactions with equilibrium dissociation constants (*K*_D_) similar to those of glycan-lectin interactions. This discovery was followed by another study by the same group, focused on the glycan-mediated adhesion of the bacterial species *Neisseria meningitidis* to human host cells. Their results, which show that glycans from the bacterial cells are able to interact with glycan structures in host cell through high-affinity bonds formed during different stages of meningococcal disease, open new paths for developing both preventive and therapeutic strategies [140,141]. A later study by O’Riordan et al. showed that glycans from several bacterial pathogens are able to interact with the glycan chains of lactoferrin, a protein present in bovine milk, although in this case no indications on the affinity of these bonds are given [142]. Finally, the body of evidence documenting the importance of glycan-glycan interactions for bacterial adhesion was recently extended by a study on the adhesion of *Shigella* to human lymphocytes [143]. Whereas Day and co-workers revealed the importance of glycan-glycan interactions in mediating binding of *Shigella* to host epithelial cells [139], this resent study by Belotserkovsky and co-workers provides evidence that glycan-glycan interactions are critical for *Shigella* pathogenesis by driving selective interactions with immune cells [143]. More precisely, they show that sialylated glycosphingolipids interact with the polysaccharide moiety of LPS, the major bacterial surface antigen, thus promoting *Shigella* binding to T cells. 

**Figure 4:**
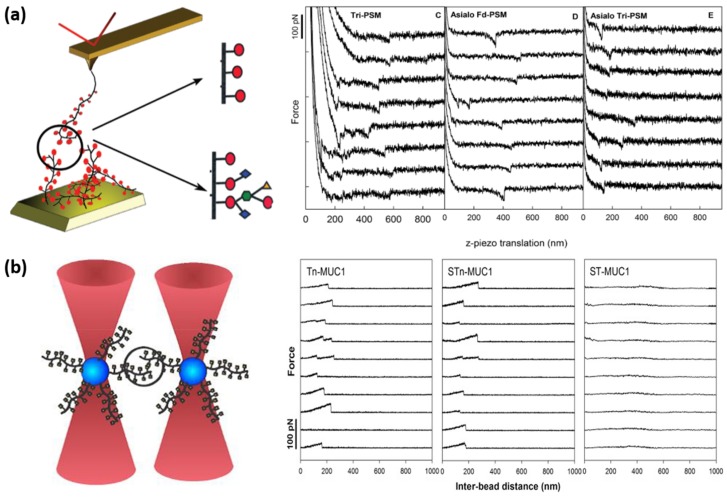
Glycan-glycan interactions probed by AFM and OT. (**a**) AFM schematic representation and force curves obtained when probing the self-interactions between AFM tips and mica surfaces functionalized with pig submaxillary mucin (PSM) harboring three different glycan decorations (Tri-PSM, Asialo Fd-PSM and Asialo Tri-PSM). Reprinted with permissions from [152]. (**b**) OT schematic representation and force curves obtained when probing the self-interactions between two polystyrene beads functionalized with mucins MUC1 harboring different glycan decorations (Tn-MUC1, STn-MUC1 and ST-MUC1). Reprinted with permissions from [154].

In these studies, a range of experimental techniques are used to determine the existence of the glycan-glycan interactions, including traditional techniques such as flow cytometry and fluorescence microscopy. Glycan microarrays and SPR are also used in other studies to quantify the kinetic constants of carbohydrate-carbohydrate interactions [144]. Other techniques that have been used to quantify glycan-glycan interactions include transmission electron microscopy (TEM) [145], nuclear magnetic resonance (NMR) [146] using carbohydrate-functionalized gold nanoparticles and glycolipid Langmuir monolayers [147] as well as cell adhesion assays using carbohydrate-coated silica nanoparticles [148]. Although neither AFM nor OT have so far been used to quantify glycan-glycan interactions involved in bacterial adhesion, these force probes are suitable tools for quantification of such weak interactions [144]. Indeed, both AFM and OT have been successfully used for the quantification of glycan-glycan interactions occurring between sponges [149] or mammalian cells [150]. Cell aggregation in the marine sponge *Microciona prolifera* is mediated by an aggregation factor (MAF) which contains a proteoglycan domain itself composed of sulfated disaccharides as well as pyruvated trisaccharides. A first study in 1999 showed using AFM that proteoglycans from this aggregation factor are able to self-interact through Ca^2+^ dependent interactions. The authors functionalized AFM tips and surfaces with proteoglycans and performed SMFS experiments. Their results are the first ones showing the role of glycan-glycan interactions in the self-adhesion of *M. prolifera* cells. Following this work, AFM was used to identify the oligosaccharides present on the proteoglycan that was responsible for its self-interactions. This study, published in 2009 by Carvalho de Souza et al. [151], also used functionalized AFM tips and surfaces, but this time with sulfated disaccharides or pyruvated trisaccharides, and clearly showed that only disaccharides are able to form homotypic glycan-glycan bonds, with an adhesion force of approximately 30 pN at the interval of force loading rates used in this study. In another study, interaction forces between individual Lewis^X^ antigen molecules (Le^X^), a trisaccharide found in human cancerous cells, were probed using AFM and Le^X^-functionalized AFM tips and surfaces [150]. This antigen was found to self-interact with a binding strength of approximately 20 pN at the force loading rate used in the study. Finally, the most recent example of glycan-glycan interactions measured by AFM was provided by Haugstad and co-workers [152,153]. In these studies, the authors used functionalized AFM tips and surfaces to measure the strength and lifetime of self-association between single pairs of well-defined mucins, characterized by their different carbohydrates decoration patterns [152]. Their results, some of which are presented in Figure 4a, showed that both the probability of self-association of the mucins, and the strength of their interactions (27–50 pN) was dependent on the glycan decoration that they harbor, thus highlighting the role of glycan-glycan interactions in these intermolecular recognition processes. Altogether, these AFM studies demonstrate the ability of SMFS experiments to measure the forces involved in glycan self-interactions. However, while the force detection limit of AFM is around 20 pN, which corresponds to the range of the forces described in these examples, OT feature a higher force sensitivity of approximately 0.5 pN. Thus, OT should be better suited to quantify these type of interactions. To date, only one example shows the utility of this technique to measure glycan-glycan interactions. In 2016, Haugstad et al. showed that OT could be used to measure the adhesion forces between self-associating mucins presenting particular glycan structures [154]. The experimental data provided in the paper (Figure 4b) allowed the authors to conclude that a particular glycan structure, the *N*-acetylgalactosamine (GalNAc), was responsible for the self-interactions observed for the mucins. 

This literature documents the relevance of glycan-glycan interactions for cell adhesion, in line with Hakomoris pioneering work and the concept named glycosynapse [155], but these interactions are poorly studied in the context of bacterial adhesion. We hypothesize that glycan-glycan interactions might be important also for the adhesion of bacterial cells to mucins found in the human gut, and future studies along these lines would thus contribute to the understanding of the gut microbiota. While a wide range of techniques can be used to study such interactions, AFM and OT are well-suited tools, and this field is thus in the future likely to benefit from the capabilities of these force probes.

### 3.5. The Importance of Glycan Patches for Bacterial Adhesion 

Both lipids and proteins form domains on biological surfaces characterized by increased surface density. Such domains are called rafts in the case of lipids [156,157], and oligomers in the case of proteins [158]. The ability of proteins to aggregate is well known in biology, and includes both amyloid aggregation [159,160] and the formation of complex structures responsible for complex functions. Usually the clustering is used to create a new function. Having that in mind, it is conceptually logical that aggregates, oligomers of glycoproteins or rafts of glycosphingolipids, form glycan surface clusters. Furthermore, carbohydrate mediated interactions are stabilized by hydrogen bonds or van der Waals interactions and are characterized by low affinity bonds. The biological relevance is often achieved by the combined action of many single interactions. In this regard, glycan clustering would facilitate the formation of multiple interactions. 

Cohen and Varki [161] reviewed the role of saccharide clustering in the modulation of glycan recognition. They focused on sialic acids [162] and the examples described in their review include experimental data providing indirect evidence for the role of saccharide clustering in Malarial merozoite recognition of clustered sialoglycans on erythrocytes glycophorins [163]. Cohen and Varki argue that “*it is likely that glycan microdomains are mostly stabilized by carbohydrate–carbohydrate interactions, which are too weak to withstand the standard biochemical methods used to study protein–protein interactions (e.g., immunoprecipitation).*” The later evidence for the ability of mucins of varying glycosylation pattern to form interactions, as provided using both AFM [152,153] and OT [154], strengthens this hypothesis.

**Figure 5:**
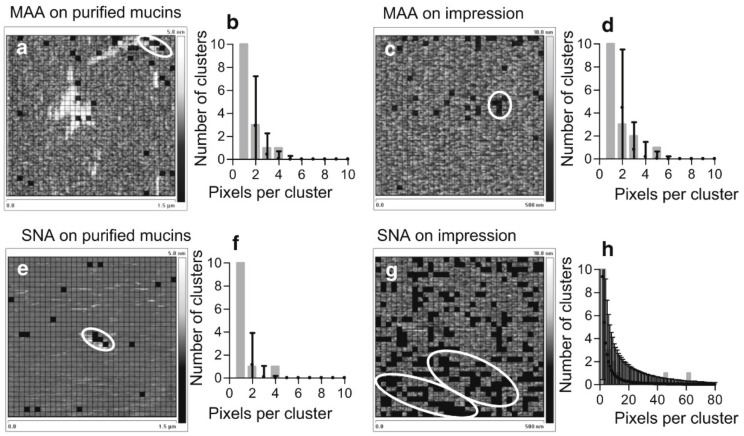
Glycan clustering probed by AFM surface topography and affinity mapping. AFM tips were functionalized with lectins *Maackia amurensis* (MAA) and *Sambucus nigra* (SNA), and the interactions with their respective receptors *α*-2,3 and *α*-2,6 *N*-acetylneuraminic (sialic) acids, present at the surface of normal human conjunctival mucins (NHC cells) or ocular cytological impressions, were probed. Force maps present height data overlaid with grids showing the location of specific interactions (solid squares) indicating the location of sialic acids: (**a**) MAA on single molecules, (**c**) MAA on impressions, (**e**) SNA on single molecules, and (**g**) SNA on impressions. The corresponding graphs (**b**, **d**, **f**, and **h**) show the expected number of neighboring interactions (mean ± 3 SD) in a simulated random distribution of equal size. The observed numbers of neighboring interactions are presented as gray bars. These are considered clustered if their number exceeds the mean + 3 SD of the simulated data and are circled in white on the force maps. Reprinted with permissions from [165].

The clustering of glycans on biological surfaces is not well described in the literature. This is most likely, as pointed out by Cohen and Varki, due to the lack of analytical techniques able to measure and localize weak interactions and unstable surface structures. The evidence for the existence of glycan clustering includes a study in which adjacent goblet cells have been observed to produce mucins deviating in their glycosylation pattern, possibly leading to clusters of particular glycosylation patterns [164]. Such clusters were later observed at mucin coated ocular surfaces using AFM [165]. In this interesting study, AFM was used to obtain force maps of human preocular mucous and purified ocular mucins by probing and locating the interactions between tip-tethered lectins *Maackia amurensis* and *Sambucus nigra* and their respective receptors, α-2,3 and α-2,6 *N*-acetylneuraminic (sialic) acids [165]. The force maps obtained, showed in Figure 5, present evidence that, in contrast to data from purified mucin molecules (Figure 5a,b,e,f), the preocular gels feature numerous large clusters (19,000 ± 4000 nm^2^) of α-2,6 sialic acids (Figure 5g,h), but very few small clusters (2000 ± 500 nm^2^) of α-2,3 epitopes (Figure 5c,d). This indicates that mucins, which are rich in α-2,3 sialic acids, are only partially exposed at the surface of the mucous gel. These results then led the authors to conclude that microorganisms that recognize α-2,3 sialic acids will encounter only isolated ligands, and the adhesion of other microorganisms will be enhanced by large islands of neighboring α-2,6 sialic acids. The use of AFM thus unveiled an additional level of mucosal surface heterogeneity, more precisely a distribution of pro- and antiadhesive sialic acids that protect underlying epithelia from viruses and bacteria. Such heterogeneity in the distribution of glycans on biological surfaces is likely not restricted to ocular surfaces, but is more widespread. This hypothesis is strengthened by the indirect evidence of sialic acid clustering provided for other systems, including the capsular polysaccharide of the bacterium (*Streptococcus* group B type III) [166]. Antibodies directed against the capsule are able to bind to it provided the bacterium is intact but unable to recognize the purified capsular polysaccharide or any comparable glycan on a microarray [166]. This is interpreted as indirect evidence for saccharide clusters on the bacterial capsule. 

A recent addition to the body of evidence documenting the importance of glycan clustering for bacterial adhesion focuses on the pathogen-recognition receptor DC-SIGN [167]. This C-type lectin receptor is highly expressed at the membrane of antigen presenting dendritic cells, where it is organized in nanoclusters and binds to different viruses, bacteria and fungi. In this paper, the involvement of the *N*-glycans of DC-SIGN expressing cells on pathogen binding strengthening when interacting with Candida fungal cells is investigated by AFM-assisted single cell-pathogen adhesion measurements. For that, *C. albicans* cells were attached to tipless AFM cantilevers that were coated by concanavalin A. The functionalized cantilevers were then brought into contact with cells expressing DC-SIGN. Cell stiffening during pathogen binding was strongly impaired in experimental series where DC-SIGN mutants lacking the *N*-glycans were used, and also upon blocking of glycan-mediated lateral interactions. The observed importance of the *N*-glycosylation of a CLR for pathogen recognition led the authors to propose *N*-glycan as future emerging targets in anti-microbial therapies, while also conveying novel concepts to the cell adhesion and mechano-biological fields.

### 3.6. Comparison of the Strength of Bacterial Surface Adhesion with Single Molecular Pair Interactions

Table 1 presents a number of studies in which AFM or OT have been used to quantify the forced rupture of protein—glycan or glycan—glycan interactions. In some of the studies the molecules were immobilized onto surfaces, in others one of the interacting partners where present on the surface of a bacterium and the binding partner was immobilized onto the surface of either a bead or the AFM tip. A challenge encountered when attempting to compare information on rupture strength as determined in different studies, is the lack of precise information on the force loading rate used. Since the rupture force is expected to dependent on the force loading rate, the lack of precise information concerning the latter is a challenge when attempting to compare information obtained in different systems. For many of the studies cited in Table 1, the force loading rate used was not clearly stated in the paper. For some of these studies, we have estimated a rupture force based on information provided concerning the spring constant as well as the separation speed used when bringing the two surfaces onto which the molecular binding partners were immobilized, apart. This approach is problematic since it does not take into account the elasticity of the linker molecules or interacting polymers [21]. Additionally, for some of these calculations, the apparent spring constant as provided from the cantilever manufacturers was used due to lack of information on the calibrated values. This has likely introduced substantial error in the estimated forces due to the known deviation between the actual spring constant and the nominal spring constant. The need for careful calibration of the spring constant of AFM cantilevers, using optimized protocols, is well documented in the literature [168]. Future studies of adhesive interactions underlying bacterial surface adhesion would benefit from increased focus on reliable determination of force loading rates and this information should be included in the publications. Such information would facilitate the comparison of results obtained in different studies.

**Figure 6:**
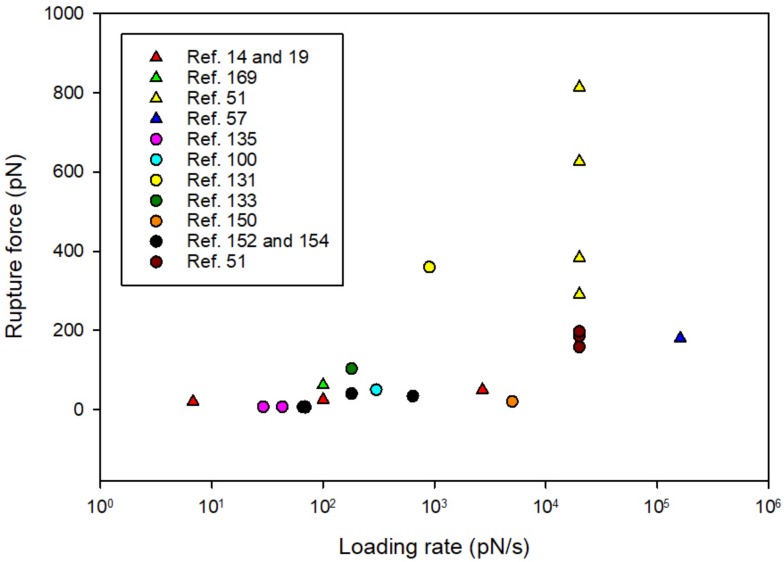
Comparison of the strength of bacterial surface adhesion as well as molecular pair interactions. The quantitative data presented in the figure is extracted from the papers presented in Table 1. The quantitative data extracted from studies on single bacterium—substrate interactions are presented using triangles whereas the data on single molecule interactions are presented using circles.

Despite the challenges described above, the available literature does provide insight into the strength of protein—glycan and glycan—glycan interactions underlying bacterial adhesion. Figure 6 is a graphical presentation of the information provided in Table 1 concerning the adhesion force of the different interactions as a function of loading rate. The figure reveal that the results provided by different groups and on different molecular pairs fall on a line where the rupture force increased with increasing force loading rate, as expected from the theory. Some interesting deviation from the linear trend are observed. The mucus-binding protein (MUB) show significantly stronger binding to mucin than what is observed for MGL [170] and also SBA [133]. This is in line with the structure and proposed multivalent binding mechanism of this large multi-repeat cell–surface adhesion, which is proposed to maximizes interactions with the mucin glycan receptors through its long and linear multi-repeat structure, potentiating the retention of bacteria within the outer mucus layer [131]. Furthermore, the binding strength observed between *H. pylori* BabA and the Le^b^ is similar in strength to the SpaC pili-protein—mucin interaction and also the SBA-mucin interaction. The slightly higher strength of the latter might be explained by SBA being a tetrameric lectin with one carbohydrate binding site per subunit [170]. This comparison support the conclusion of the authors that the BabA-Le^b^ rupture events observed reflect single-molecular pair interactions [14,106]. On the other hand, the strong interaction observed between *S. aureus* and surfaces coated with either the complement system proteins or laminin, indicate high degree of multiplicity. This multiplicity is reduced when immobilizing the purified *S. aureus* collagen binding protein (Cna) to the probe surface instead of the bacterial cell [51]. Such comparisons of the adhesive forces observed between bacteria and functionalized surfaces, or relevant biological surfaces, with the strength observed for relevant single molecular pair interactions can provide insight into the multiplicity of the active adhesive interactions. The lifetime of the interactions, also provide interesting information. The bacterial adhesion that is based on multiple molecular pair interactions [51] are expected to show longer lifetimes than most of the single molecular pair based interactions studied. The limited amount of data available makes a comparison challenging at this stage. 

## 4. Concluding Remarks 

Glycobiology is a field undergoing spectacular progress, resulting in the revelation of the critical role of glycoproteins in the organization and function of eukaryotic cells. These advances have, like most other advances in biology, greatly benefited from the development and refinement of technological tools available to the researchers. We have here reviewed how the sensitive force probes AFM and OT have contributed to the recent advances in our understanding of how glycans contribute to the adhesion of bacteria to mucosal surfaces, by revealing biophysical features of the adhesive interactions underlying microbial adhesion. This adhesion is often driven by several mechanisms that act in parallel or in a consecutive manner. Studies performed using AFM and OT have provided insight into the relative importance of each of these mechanisms under different environmental conditions. The literature contain examples where these force probes have provided insight into both pili-based and non-pili based attachment to mucosal surfaces. Many of these adhesion events occur in nature under conditions of applied external force, due to the bacteria being transported in a liquid flow. The ability to study the adhesion events under different conditions of applied force, through the realization of dynamic force spectroscopy measurements, is thus relevant and important. Furthermore, the high sensitivity of these force probes makes them well suited to study weak glycan-based interactions. Both AFM and OT have already proven their applicability for quantification of both protein-glycan and bacteria-surface interactions. Furthermore, the importance of glycan-glycan interactions for bacterial adhesion to biological surfaces, including the surface of human cells, has recently been documented. AFM and OT have not yet been applied to address this mechanism of bacterial adhesion, despite their clear application potential for such studies. Last but not least, AFM has contributed to the growing body of evidence documenting that glycan patches represent a degree of complexity in cell-cell and host-cell recognition and dialog that needs to be taken into account. We hope the capabilities of these experimental techniques as explained in this review and in the cited literature inspire new studies of glycan-based interactions and the mechanisms underlying bacterial surface adhesion to both mucosal and other surfaces.

## Figures and Tables

**Table 1:** The strength of protein—glycan and glycan—glycan molecular pair interactions determined using sensitive force probes.

	Reference	System Studied	Force Probe	Rupture Force (pN)	Loading Rate (pN/s)	k_off_ (1/s)
**Single bacterium-substrate interactions**	**Protein-glycan interactions**					
	Björnham [14]	*H. pylori* BabA—Le^b^	OT	25 ± 1.5	100	N/A
	Björnham [19]	*H. pylori* BabA—Le^b^	OT	20 * 50 *	6.8 2700	0.86 ± 0.07
	Beaussart [169]	*P. aeruginosa* pili—Pneumocyte	AFM	62 ± 11	100	N/A
	Simpson [11]	*S. aureus*—fibrinogen *S. aureus*—fibronectin	OT	20 25	N/A N/A	N/A
	Le [57]	*L. lactis*—PGM	AFM	180 ± 4	161,250	N/A
	**Protein-protein interactions**					
	Valotteau et al. [51]	*S. aureus*—complement system protein (C1q)	AFM	291 ± 97 (JPK instrument) 384 ± 48 (Bruker instrument)	~20,000 ^#^	N/A
		*S. aureus*—Laminin	AFM	814 ± 179 (JPK instrument) 627 ± 122 (Bruker instrument)	~20,000 ^#^	N/A
**Single molecule interactions**	**Protein-glycan interactions**					
	Hadjialirezaei [135]	MUC1(Tn)—MGL	OT	6.8 ± 0.8	29 ± 2	2.0
		MUC1(STn)—MGL	OT	7.1 ± 1.1	43 ± 3	3.3
	Tripathi [100]	SpaC pili-protein—mucin	AFM	50	300 *	0.05
	Gunning [131]	MUB—mucin	AFM	380–342	900 *	N/A
	Sletmoen [133]	SBA—PGM	AFM	103	180	0.76 ± 0.09
	**Glycan-glycan interactions**					
	Tromas [150]	Le^x^—Le^x^ self-interaction	AFM	20 ± 4	~5000	N/A
	Haugstad [152]	Tn-PSM—Tn-PSM	AFM	34	640	1.2 ± 0.27
		Fd-PSM—Fd-PSM		40	180	0.64 ± 0.8
	Haugstad [154]	Tn-PSM—Tn-PSM	OT	7.2	69	3.8
		Tri-PSM—Tri-PSM		5.6	69	5.0
		STn-OSM—STn-OSM		6.2	66	6.2

* Estimated based on graphical material provided in the cited publications; ^#^ Estimated based on information provided in the paper related to nominal spring constant and tip retraction speed.
